# Spontaneous Spinal Cord Infarction Presenting as Chest Pain: A Case Report

**DOI:** 10.7759/cureus.90451

**Published:** 2025-08-19

**Authors:** Kenjiro Yamane, Tomotaka Takanosu, Tomohiro Matsumura, Chikara Yonekawa, Takashi Mato

**Affiliations:** 1 Emergency and Critical Care Medicine, Jichi Medical University, Shimotsuke, JPN

**Keywords:** acute myelopathy, physical diagnosis, spinal cord disease, spinal cord ischemia, stroke

## Abstract

A 66-year-old woman with a history of hypertension presented to the emergency department with sudden-onset middle anterior chest pain. Initial evaluations, including general examination, electrocardiography, and contrast-enhanced computed tomography (CT), did not reveal the cause. However, on subsequent physical examination, mild weakness in the right lower limb was noted, which prompted MRI and ultimately led to the diagnosis of spinal cord infarction (SCI).

Diagnosing SCI is often challenging, but, in this case, careful clinical examination was key. This case underscores the importance of repeated and thorough clinical assessments. Although no established treatment exists, underdiagnosis may be one reason. Accurate identification of SCI can improve understanding of its pathophysiology and help guide the development of effective therapies.

## Introduction

Spinal cord infarction (SCI) is a well-recognized cause of acute myelopathy. Typical symptoms include sudden or acute onset of bilateral lower limb weakness and sensory deficits. However, such symptoms may not always be evident early in the disease course, with approximately 20% of cases initially presenting with unilateral lower limb paralysis and paresthesia [1]. Additionally, around 70% of patients report back pain as an initial symptom [2,3], although pain in other regions, such as the limbs, has also been described [1]. Few reports have examined the distribution of pain locations, and cases in which chest pain is the prominent initial symptom of SCI are exceedingly rare [4]. Although the exact incidence is unknown and therapies are not established, underdiagnosis has been cited as one possible reason for this [5]. We report a case of SCI in which the patient initially presented with chest pain, an atypical symptom, and was diagnosed only after subtle right lower limb weakness was detected. This case highlights the importance of repeated, thorough clinical examinations, as improving diagnostic accuracy can help guide the development of effective treatments.

## Case presentation

A 66-year-old woman with a history of hypertension presented to the emergency department with a sudden-onset, central, squeezing chest that began upon waking. She arrived at the hospital approximately two hours after symptom onset.

On admission, her blood pressure was 252/124 mmHg in the right arm and 219/118 mmHg in the left arm, heart rate was 72 beats per minute, respiratory rate was 20 breaths per minute, body temperature was 36.2°C, and oxygen saturation was 99% on room air. No cold sweats were observed. Heart sounds were normal, with no murmurs. Radio-radial and radio-femoral delays were not present. Breath sounds were clear bilaterally. No rash or abnormal sensation was noted on the chest. The abdomen was soft and non-distended, with no rebound tenderness or guarding. Laboratory findings are shown in Table 1.

**Table 1:** Blood test results WBC: white blood cell, Hb: hemoglobin, Plt: platelet, PT-INR: prothrombin time-international normalized ratio, APTT: activated partial thromboplastin time, CRP: C-reactive protein, TP: total protein, Alb: albumin, BUN: blood urea nitrogen, Cr: creatinine, T-Bil: total bilirubin, AST: aspartate aminotransferase, ALT: alanine aminotransferase, LDH: lactate dehydrogenase, ALP: alkaline phosphatase, γ-GTP: γ-glutamyl transpeptidase, T-Chol: total cholesterol, TG: triglycerides, HDL-C: high-density lipoprotein cholesterol, CK: creatine kinase, Na: sodium, K: potassium, Ca: calcium, Glu: serum glucose, TnT: troponin T, HbA1c: hemoglobin A1c.

Parameter	Result	Reference range
WBC	9 x 10^3^ /μL	3.3-8.6 x 10^3^/μL
Hb	14.6 g/dL	11.6-14.8 g/dL
Plt	295 x 10^3^ /μL	158-348 x 10^3^ /μL
PT-INR	1.00	0.85-1.15
APTT	27.6 sec	22.4-37.4 sec
D-dimer	1.1 μg/mL	<1 μg/mL
CRP	1.16 mg/dL	0.00-0.14 mg/dL
TP	8.4 g/dL	6.6-8.1 g/dL
Alb	4.3 g/dL	4.1-5.1 g/dL
BUN	12 mg/dL	8-20 mg/dL
Cr	0.62 mg/dL	0.46-0.79 mg/dL
T-Bil	0.74 mg/dL	0.40-1.50 mg/dL
AST	24 U/L	13-30 U/L
ALT	17 U/L	7-23 U/L
LDH	199 U/L	124-222 U/L
ALP	104 U/L	38-113
γ-GTP	20 U/L	9-32 U/L
T-Chol	259 mg/dL	142-248 mg/dL
TG	259 mg/dL	30-117 mg/dL
HDL-C	70 mg/dL	48-103 mg/dL
Lipase	38 U/L	13-49 U/L
CK	97 U/L	41-153 U/L
Na	139 mmol/L	138-145 mmol/L
K	3.7 mmol/L	3.6-4.8 mmol/L
Ca	9.4 mg/dL	8.8-10.1 mg/dL
Glu	125 mg/dL	73-109 mg/dL
TnT	0.007 ng/mL	<0.014 ng/mL
HbA1c	6.50%	4.9-6.0%

Cerebrospinal fluid (CSF) findings are shown in Table 2.

**Table 2:** CSF analysis CSF: cerebrospinal fluid, RBC: red blood cell, TP: total protein, Glu: glucose.

Parameter	Result
Cell count	2/μL
RBC	100/μL
Appearance	Clear and colorless
TP	38 mg/dL
Glu	61 mg/dL

Electrocardiography and chest radiography showed no abnormalities. Transthoracic echocardiography revealed normal cardiac structure and function. Contrast-enhanced computed tomography (CT), performed to rule out structural causes of sudden-onset chest pain, including aortic dissection, also showed no abnormalities.

After the initial evaluation, the patient reported difficulty walking and mild weakness in the right lower extremity. Manual muscle testing (MMT) revealed right lower limb weakness with a score of 4/5. There were no symptoms in the upper limbs, and sensation and tendon reflexes were normal. Suspecting a brain or spinal cord lesion, the patient was admitted for further investigation.

**Figure 1:**
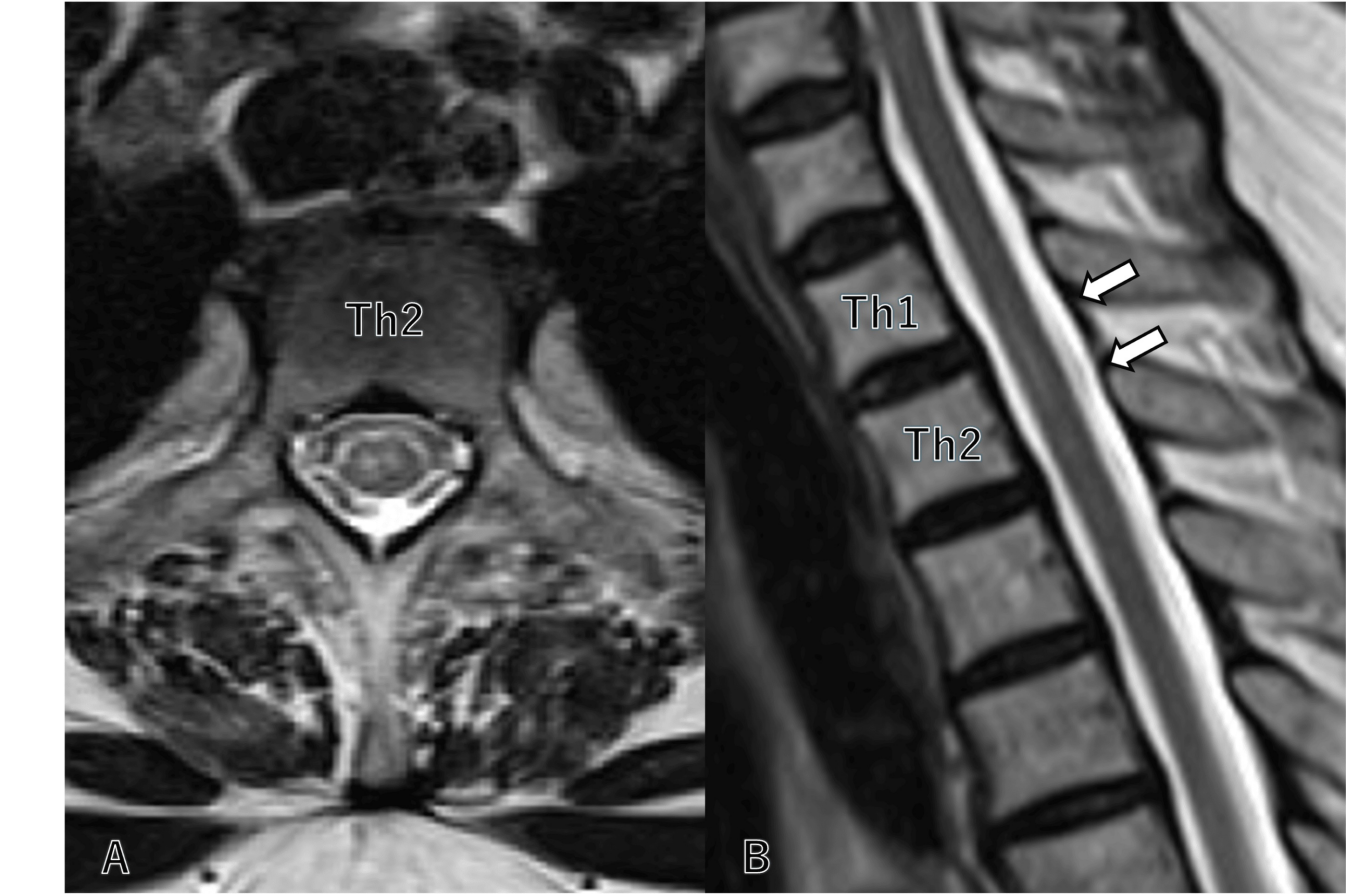
MRI performed 10 hours after symptom onset T2-weighted images show high signal intensity and diffusion restriction in the anterior horns (A) of the spinal cord at the T1-T2 vertebral level (B).

Brain magnetic resonance imaging (MRI) showed no abnormalities suggestive of ischemic or neurodegenerative disease. Thoracic spinal MRI, performed 10 hours after symptom onset, revealed high signal intensity on T2-weighted images and diffusion restriction in the anterior horn of the spinal cord at the Th1/2 vertebral level, consistent with SCI (Figure 1).

After excluding other differential diagnoses and consulting a neurologist, a diagnosis of SCI was established. On the day after admission, the patient developed right-sided sensory loss below the nipple level, with further deterioration of right lower limb strength to an MMT score of 1/5, accompanied by a positive Babinski reflex. Treatment with aspirin and immunosuppressants was initiated. However, no improvement was observed by the second day of admission, and she was transferred to another hospital for rehabilitation on day 15.

## Discussion

SCI is caused by occlusion or embolism of the arteries supplying the spinal cord. Although the exact incidence is unknown, SCI is extremely rare, accounting for less than 1% of all strokes [5]. Typical symptoms include acute-onset motor weakness and sensory disturbances corresponding to the affected spinal cord level. However, depending on the region involved, patients may present with a variety of symptoms, including dizziness or unsteadiness, which may reflect posterior column involvement [6].

Several reports indicate that 53%-83% of patients experience persistent gait disturbances, and 2%-10% either die or remain bed-bound at the time of death. Additionally, 10% of patients rely on a cane, 15% use a walker, and 26% require a wheelchair. These findings highlight the significant impact of SCI on functional ability and overall prognosis [1,7]. However, aggressive rehabilitation can result in favorable outcomes, with 47% of patients able to walk without a gait aid [1]. Furthermore, 14%-16% of patients initially diagnosed with transverse myelitis are later reclassified as having SCI, suggesting that idiopathic SCI may be underdiagnosed. Clinician awareness is therefore essential for timely diagnosis [1].

While back or limb pain is reported in approximately 72% of patients at symptom onset, reports of chest pain are exceedingly rare [1]. Among patients with cervical spondylosis, a condition known as cervical angina can present with sudden chest pain that mimics angina pectoris [8]. Although about 30% of SCIs occur at the C6-C7 level, upper thoracic segments may also produce chest pain depending on the pattern of nerve distribution. In this case, the chest pain was likely neuropathic, corresponding to the affected dermatomes, rather than being directly caused by spinal artery occlusion.

Diagnosis of SCI is based on clinical symptoms, imaging findings, and exclusion of other conditions. Typical MRI features of anterior spinal artery infarction include T2 hyperintensity and diffusion restriction localized to the anterior horns, often described as the “snake-eye” or “owl-eye” appearance. However, one study reported that the sensitivity of spinal MRI for detecting SCI was 72.4%, and only around 25% within the first six hours. Sensitivity peaks between 24 and 72 hours [9]. Therefore, if SCI is suspected based on history and physical examination, repeat imaging is essential. Additionally, as in this case, 32% of patients with SCI develop motor neurological deficits more than one hour after symptom onset. Thus, even in the absence of early motor paralysis, repeated neurological assessments are crucial [1].

A definitive treatment for SCI has not yet been established. Proposed strategies include avoiding hypotension, anticoagulation, and systemic corticosteroids. Although avoiding hypotension is commonly practiced, optimal blood pressure targets have not been defined, and prospective studies are lacking [1]. A systematic review reported that corticosteroids were administered in approximately 41% of cases prior to diagnosis, often to differentiate SCI from myelitis. However, there is insufficient evidence to support corticosteroid use in SCI, and caution is advised due to potential adverse effects. Antiplatelet therapy is often employed empirically, but its efficacy has not been demonstrated [2]. In this case, a combination of immunosuppressive and antiplatelet therapies was administered; however, no improvement in paralysis was observed. One reason for the lack of established treatment may be the rarity of SCI. Many cases may be underdiagnosed, particularly when patients present with atypical symptoms such as chest pain. To develop effective therapies and improve neurological outcomes, clinicians need to recognize SCI and include it in the differential diagnosis.

## Conclusions

Although chest pain is an uncommon presentation of SCI, recognition of subtle lower limb weakness in this case enabled an accurate diagnosis. This case highlights the importance of considering SCI even in patients with atypical symptoms such as chest pain. In instances of sudden-onset chest pain with no identifiable cause, SCI should be included in the differential diagnosis, and physical findings should be reassessed repeatedly. Accurate identification of these underdiagnosed cases can improve understanding of the pathophysiology and ultimately support the development of effective treatments.
